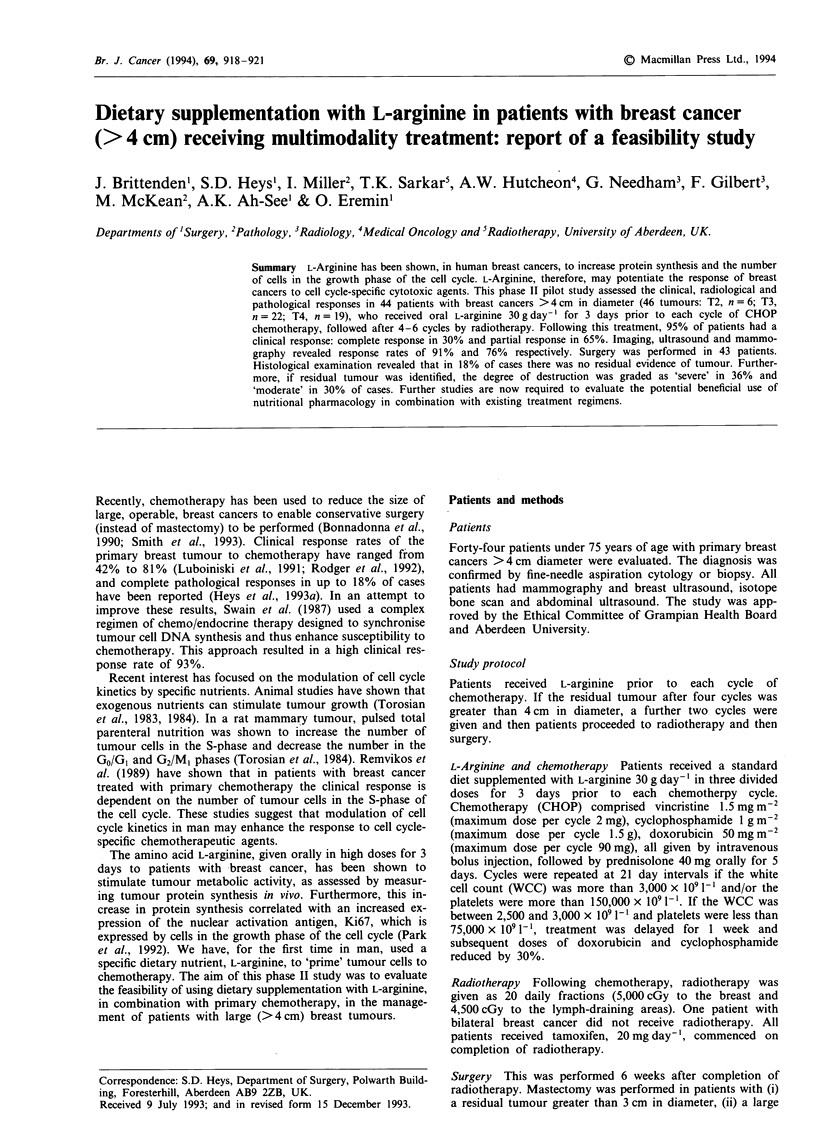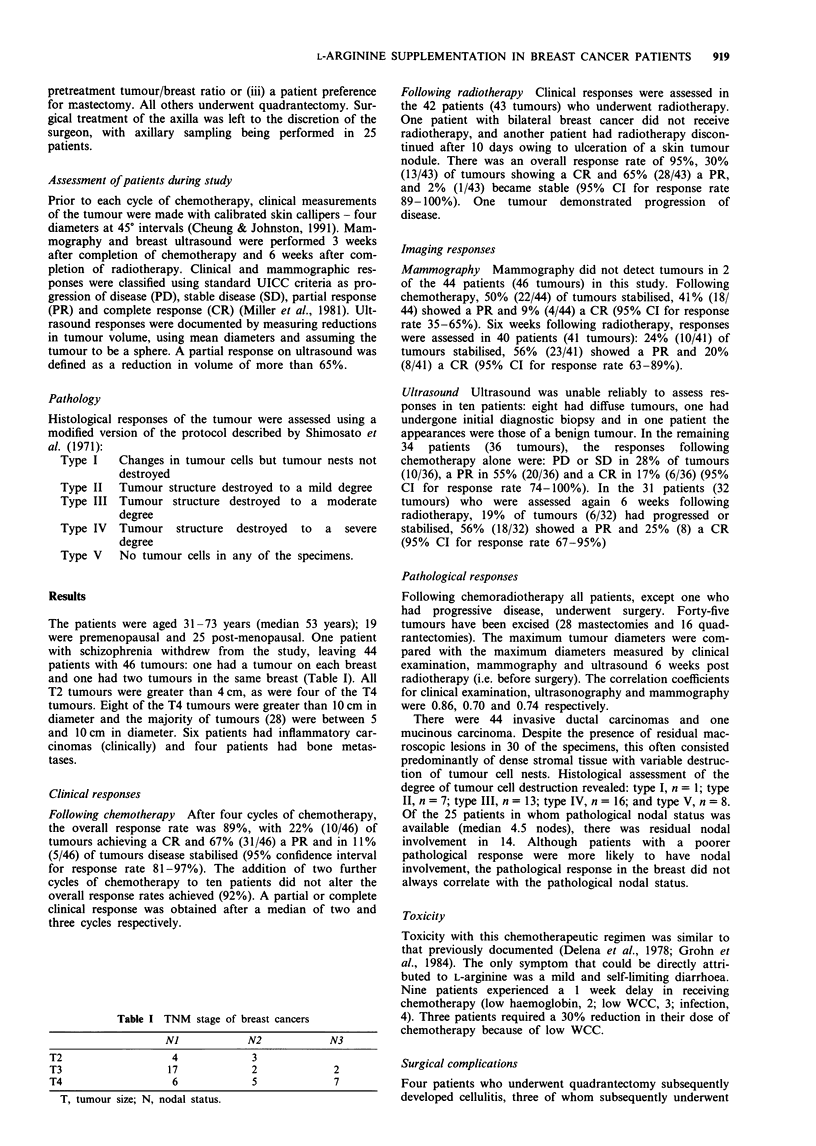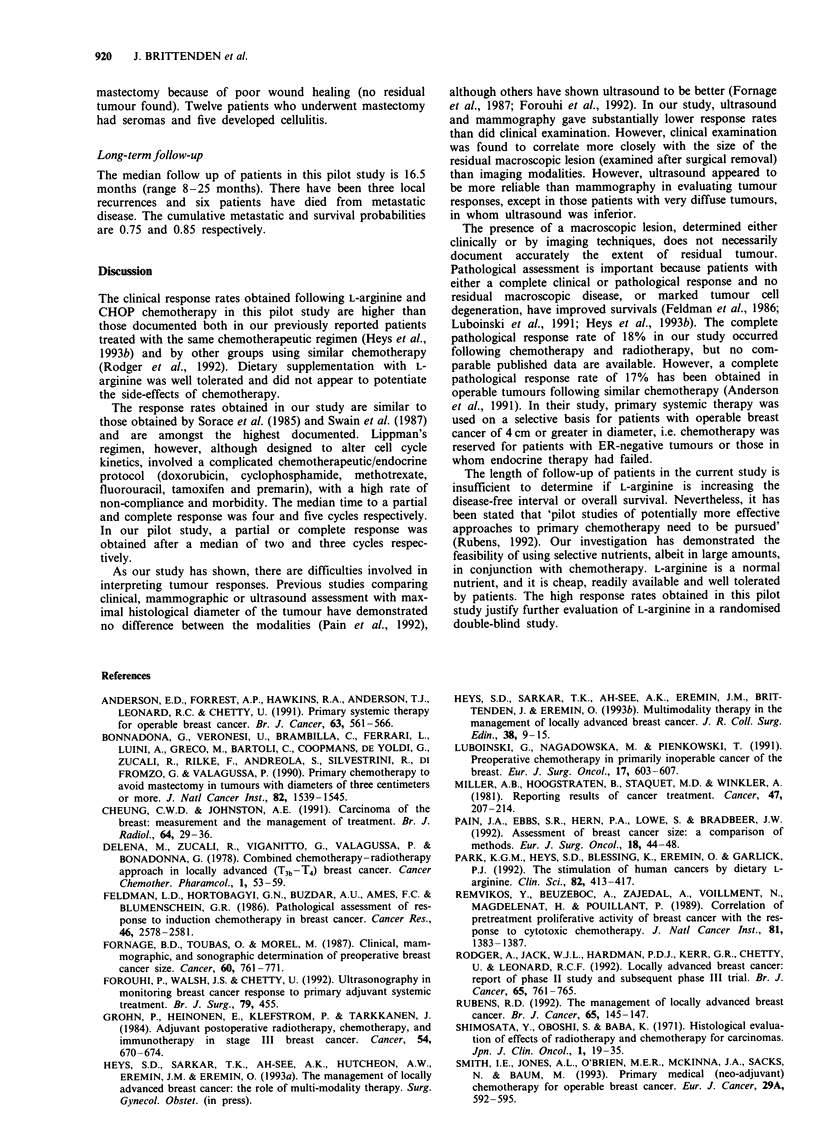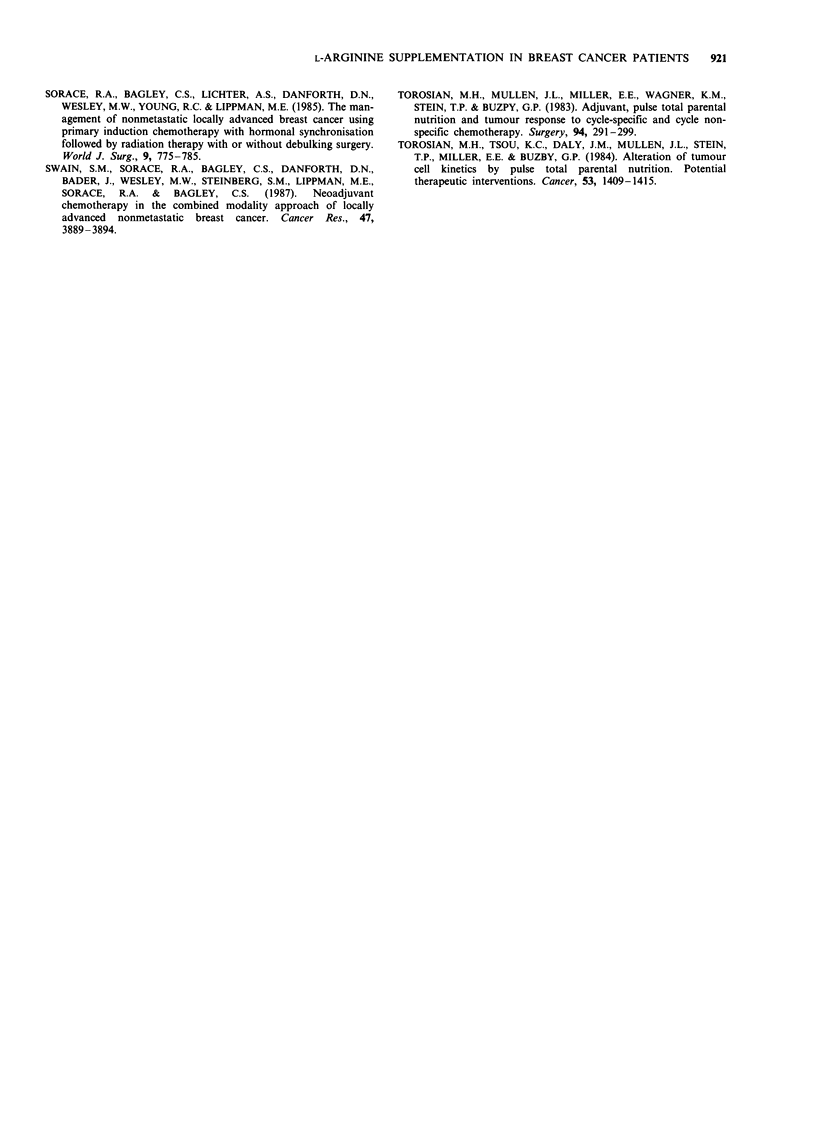# Dietary supplementation with L-arginine in patients with breast cancer (> 4 cm) receiving multimodality treatment: report of a feasibility study.

**DOI:** 10.1038/bjc.1994.177

**Published:** 1994-05

**Authors:** J. Brittenden, S. D. Heys, I. Miller, T. K. Sarkar, A. W. Hutcheon, G. Needham, F. Gilbert, M. McKean, A. K. Ah-See, O. Eremin

**Affiliations:** Department of Surgery, University of Aberdeen, UK.

## Abstract

L-Arginine has been shown, in human breast cancers, to increase protein synthesis and the number of cells in the growth phase of the cell cycle. L-Arginine, therefore, may potentiate the response of breast cancers to cell cycle-specific cytotoxic agents. This phase II pilot study assessed the clinical, radiological and pathological responses in 44 patients with breast cancers > 4 cm in diameter (46 tumours: T2, n = 6; T3, n = 22; T4, n = 19), who received oral L-arginine 30 g day-1 for 3 days prior to each cycle of CHOP chemotherapy, followed after 4-6 cycles by radiotherapy. Following this treatment, 95% of patients had a clinical response: complete response in 30% and partial response in 65%. Imaging, ultrasound and mammography revealed response rates of 91% and 76% respectively. Surgery was performed in 43 patients. Histological examination revealed that in 18% of cases there was no residual evidence of tumour. Furthermore, if residual tumour was identified, the degree of destruction was graded as 'severe' in 36% and 'moderate' in 30% of cases. Further studies are now required to evaluate the potential beneficial use of nutritional pharmacology in combination with existing treatment regimens.


Br. J. Cancer (1994), 69, 918-921 © Macmillan Press Ltd., 1994

### Dietary supplementation with L-arginine in patients with breast cancer (>4 cm) receiving multimodality treatment: report of a feasibility study

J. Brittenden[1], S.D. Heys[1], I. Miller[2], T.K. Sarkar[5], A.W. Hutcheon[4], G. Needham[3], F. Gilbert[3], M. McKean[2], A.K. Ah-See[1] & O. Eremin[1]

*Departments of [1]Surgery, [2]Pathology, [3]Radiology, [4]Medical Oncology and [5]Radiotherapy, University of Aberdeen, UK.*

**Summary** L-Arginine has been shown, in human breast cancers, to increase protein synthesis and the number of cells in the growth phase of the cell cycle. L-Arginine, therefore, may potentiate the response of breast cancers to cell cycle-specific cytotoxic agents. This phase II pilot study assessed the clinical, radiological and pathological responses in 44 patients with breast cancers >4 cm in diameter (46 tumours: T2, $n = 6$; T3, $n = 22$; T4, $n = 19$), who received oral L-arginine 30 g day$^{-1}$ for 3 days prior to each cycle of CHOP chemotherapy, followed after 4-6 cycles by radiotherapy. Following this treatment, 95% of patients had a clinical response: complete response in 30% and partial response in 65%. Imaging, ultrasound and mammography revealed response rates of 91% and 76% respectively. Surgery was performed in 43 patients. Histological examination revealed that in 18% of cases there was no residual evidence of tumour. Furthermore, if residual tumour was identified, the degree of destruction was graded as 'severe' in 36% and 'moderate' in 30% of cases. Further studies are now required to evaluate the potential beneficial use of nutritional pharmacology in combination with existing treatment regimens.

Recently, chemotherapy has been used to reduce the size of large, operable, breast cancers to enable conservative surgery (instead of mastectomy) to be performed (Bonnadonna *et al.*, 1990; Smith *et al.*, 1993). Clinical response rates of the primary breast tumour to chemotherapy have ranged from 42% to 81% (Luboiniski *et al.*, 1991; Rodger *et al.*, 1992), and complete pathological responses in up to 18% of cases have been reported (Heys *et al.*, 1993*a*). In an attempt to improve these results, Swain *et al.* (1987) used a complex regimen of chemo/endocrine therapy designed to synchronise tumour cell DNA synthesis and thus enhance susceptibility to chemotherapy. This approach resulted in a high clinical response rate of 93%.

Recent interest has focused on the modulation of cell cycle kinetics by specific nutrients. Animal studies have shown that exogenous nutrients can stimulate tumour growth (Torosian *et al.*, 1983, 1984). In a rat mammary tumour, pulsed total parenteral nutrition was shown to increase the number of tumour cells in the S-phase and decrease the number in the $G_0/G_1$ and $G_2/M_1$ phases (Torosian *et al.*, 1984). Remvikos *et al.* (1989) have shown that in patients with breast cancer treated with primary chemotherapy the clinical response is dependent on the number of tumour cells in the S-phase of the cell cycle. These studies suggest that modulation of cell cycle kinetics in man may enhance the response to cell cycle-specific chemotherapeutic agents.

The amino acid L-arginine, given orally in high doses for 3 days to patients with breast cancer, has been shown to stimulate tumour metabolic activity, as assessed by measuring tumour protein synthesis *in vivo*. Furthermore, this increase in protein synthesis correlated with an increased expression of the nuclear activation antigen, Ki67, which is expressed by cells in the growth phase of the cell cycle (Park *et al.*, 1992). We have, for the first time in man, used a specific dietary nutrient, L-arginine, to 'prime' tumour cells to chemotherapy. The aim of this phase II study was to evaluate the feasibility of using dietary supplementation with L-arginine, in combination with primary chemotherapy, in the management of patients with large (>4 cm) breast tumours.

Correspondence: S.D. Heys, Department of Surgery, Polwarth Building, Foresterhill, Aberdeen AB9 2ZB, UK.
Received 9 July 1993; and in revised form 15 December 1993.

### Patients and methods

### Patients

Forty-four patients under 75 years of age with primary breast cancers >4 cm diameter were evaluated. The diagnosis was confirmed by fine-needle aspiration cytology or biopsy. All patients had mammography and breast ultrasound, isotope bone scan and abdominal ultrasound. The study was approved by the Ethical Committee of Grampian Health Board and Aberdeen University.

### Study protocol

Patients received L-arginine prior to each cycle of chemotherapy. If the residual tumour after four cycles was greater than 4 cm in diameter, a further two cycles were given and then patients proceeded to radiotherapy and then surgery.

*L-Arginine and chemotherapy* Patients received a standard diet supplemented with L-arginine 30 g day$^{-1}$ in three divided doses for 3 days prior to each chemotherpy cycle. Chemotherapy (CHOP) comprised vincristine 1.5 mg m$^{-2}$ (maximum dose per cycle 2 mg), cyclophosphamide 1 g m$^{-2}$ (maximum dose per cycle 1.5 g), doxorubicin 50 mg m$^{-2}$ (maximum dose per cycle 90 mg), all given by intravenous bolus injection, followed by prednisolone 40 mg orally for 5 days. Cycles were repeated at 21 day intervals if the white cell count (WCC) was more than 3,000 × 10$^9$ l$^{-1}$ and/or the platelets were more than 150,000 × 10$^9$ l$^{-1}$. If the WCC was between 2,500 and 3,000 × 10$^9$ l$^{-1}$ and platelets were less than 75,000 × 10$^9$ l$^{-1}$, treatment was delayed for 1 week and subsequent doses of doxorubicin and cyclophosphamide reduced by 30%.

*Radiotherapy* Following chemotherapy, radiotherapy was given as 20 daily fractions (5,000 cGy to the breast and 4,500 cGy to the lymph-draining areas). One patient with bilateral breast cancer did not receive radiotherapy. All patients received tamoxifen, 20 mg day$^{-1}$, commenced on completion of radiotherapy.

*Surgery* This was performed 6 weeks after completion of radiotherapy. Mastectomy was performed in patients with (i) a residual tumour greater than 3 cm in diameter, (ii) a large pretreatment tumour/breast ratio or (iii) a patient preference for mastectomy. All others underwent quadrantectomy. Surgical treatment of the axilla was left to the discretion of the surgeon, with axillary sampling being performed in 25 patients.

*Assessment of patients during study*

Prior to each cycle of chemotherapy, clinical measurements of the tumour were made with calibrated skin callipers – four diameters at 45° intervals (Cheung & Johnston, 1991). Mammography and breast ultrasound were performed 3 weeks after completion of chemotherapy and 6 weeks after completion of radiotherapy. Clinical and mammographic responses were classified using standard UICC criteria as progression of disease (PD), stable disease (SD), partial response (PR) and complete response (CR) (Miller *et al*., 1981). Ultrasound responses were documented by measuring reductions in tumour volume, using mean diameters and assuming the tumour to be a sphere. A partial response on ultrasound was defined as a reduction in volume of more than 65%.

*Pathology*

Histological responses of the tumour were assessed using a modified version of the protocol described by Shimosato *et al*. (1971):

Type I Changes in tumour cells but tumour nests not destroyed
Type II Tumour structure destroyed to a mild degree
Type III Tumour structure destroyed to a moderate degree
Type IV Tumour structure destroyed to a severe degree
Type V No tumour cells in any of the specimens.

### Results

The patients were aged 31–73 years (median 53 years); 19 were premenopausal and 25 post-menopausal. One patient with schizophrenia withdrew from the study, leaving 44 patients with 46 tumours: one had a tumour on each breast and one had two tumours in the same breast (Table I). All T2 tumours were greater than 4 cm, as were four of the T4 tumours. Eight of the T4 tumours were greater than 10 cm in diameter and the majority of tumours (28) were between 5 and 10 cm in diameter. Six patients had inflammatory carcinomas (clinically) and four patients had bone metastases.

*Clinical responses*

*Following chemotherapy* After four cycles of chemotherapy, the overall response rate was 89%, with 22% (10/46) of tumours achieving a CR and 67% (31/46) a PR and in 11% (5/46) of tumours disease stabilised (95% confidence interval for response rate 81–97%). The addition of two further cycles of chemotherapy to ten patients did not alter the overall response rates achieved (92%). A partial or complete clinical response was obtained after a median of two and three cycles respectively.

**Table I** TNM stage of breast cancers

| | *N1* | *N2* | *N3* |
|---|---|---|---|
| T2 | 4 | 3 | |
| T3 | 17 | 2 | 2 |
| T4 | 6 | 5 | 7 |

T, tumour size; N, nodal status.

*Following radiotherapy* Clinical responses were assessed in the 42 patients (43 tumours) who underwent radiotherapy. One patient with bilateral breast cancer did not receive radiotherapy, and another patient had radiotherapy discontinued after 10 days owing to ulceration of a skin tumour nodule. There was an overall response rate of 95%, 30% (13/43) of tumours showing a CR and 65% (28/43) a PR, and 2% (1/43) became stable (95% CI for response rate 89–100%). One tumour demonstrated progression of disease.

*Imaging responses*

*Mammography* Mammography did not detect tumours in 2 of the 44 patients (46 tumours) in this study. Following chemotherapy, 50% (22/44) of tumours stabilised, 41% (18/44) showed a PR and 9% (4/44) a CR (95% CI for response rate 35–65%). Six weeks following radiotherapy, responses were assessed in 40 patients (41 tumours): 24% (10/41) of tumours stabilised, 56% (23/41) showed a PR and 20% (8/41) a CR (95% CI for response rate 63–89%).

*Ultrasound* Ultrasound was unable reliably to assess responses in ten patients: eight had diffuse tumours, one had undergone initial diagnostic biopsy and in one patient the appearances were those of a benign tumour. In the remaining 34 patients (36 tumours), the responses following chemotherapy alone were: PD or SD in 28% of tumours (10/36), a PR in 55% (20/36) and a CR in 17% (6/36) (95% CI for response rate 74–100%). In the 31 patients (32 tumours) who were assessed again 6 weeks following radiotherapy, 19% of tumours (6/32) had progressed or stabilised, 56% (18/32) showed a PR and 25% (8) a CR (95% CI for response rate 67–95%)

*Pathological responses*

Following chemoradiotherapy all patients, except one who had progressive disease, underwent surgery. Forty-five tumours have been excised (28 mastectomies and 16 quadrantectomies). The maximum tumour diameters were compared with the maximum diameters measured by clinical examination, mammography and ultrasound 6 weeks post radiotherapy (i.e. before surgery). The correlation coefficients for clinical examination, ultrasonography and mammography were 0.86, 0.70 and 0.74 respectively.

There were 44 invasive ductal carcinomas and one mucinous carcinoma. Despite the presence of residual macroscopic lesions in 30 of the specimens, this often consisted predominantly of dense stromal tissue with variable destruction of tumour cell nests. Histological assessment of the degree of tumour cell destruction revealed: type I, $n = 1$; type II, $n = 7$; type III, $n = 13$; type IV, $n = 16$; and type V, $n = 8$. Of the 25 patients in whom pathological nodal status was available (median 4.5 nodes), there was residual nodal involvement in 14. Although patients with a poorer pathological response were more likely to have nodal involvement, the pathological response in the breast did not always correlate with the pathological nodal status.

*Toxicity*

Toxicity with this chemotherapeutic regimen was similar to that previously documented (Delena *et al*., 1978; Grohn *et al*., 1984). The only symptom that could be directly attributed to L-arginine was a mild and self-limiting diarrhoea. Nine patients experienced a 1 week delay in receiving chemotherapy (low haemoglobin, 2; low WCC, 3; infection, 4). Three patients required a 30% reduction in their dose of chemotherapy because of low WCC.

*Surgical complications*

Four patients who underwent quadrantectomy subsequently developed cellulitis, three of whom subsequently underwent mastectomy because of poor wound healing (no residual tumour found). Twelve patients who underwent mastectomy had seromas and five developed cellulitis.

*Long-term follow-up*

The median follow up of patients in this pilot study is 16.5 months (range 8–25 months). There have been three local recurrences and six patients have died from metastatic disease. The cumulative metastatic and survival probabilities are 0.75 and 0.85 respectively.

### Discussion

The clinical response rates obtained following L-arginine and CHOP chemotherapy in this pilot study are higher than those documented both in our previously reported patients treated with the same chemotherapeutic regimen (Heys *et al.*, 1993*b*) and by other groups using similar chemotherapy (Rodger *et al.*, 1992). Dietary supplementation with L-arginine was well tolerated and did not appear to potentiate the side-effects of chemotherapy.

The response rates obtained in our study are similar to those obtained by Sorace *et al.* (1985) and Swain *et al.* (1987) and are amongst the highest documented. Lippman's regimen, however, although designed to alter cell cycle kinetics, involved a complicated chemotherapeutic/endocrine protocol (doxorubicin, cyclophosphamide, methotrexate, fluorouracil, tamoxifen and premarin), with a high rate of non-compliance and morbidity. The median time to a partial and complete response was four and five cycles respectively. In our pilot study, a partial or complete response was obtained after a median of two and three cycles respectively.

As our study has shown, there are difficulties involved in interpreting tumour responses. Previous studies comparing clinical, mammographic or ultrasound assessment with maximal histological diameter of the tumour have demonstrated no difference between the modalities (Pain *et al.*, 1992), although others have shown ultrasound to be better (Fornage *et al.*, 1987; Forouhi *et al.*, 1992). In our study, ultrasound and mammography gave substantially lower response rates than did clinical examination. However, clinical examination was found to correlate more closely with the size of the residual macroscopic lesion (examined after surgical removal) than imaging modalities. However, ultrasound appeared to be more reliable than mammography in evaluating tumour responses, except in those patients with very diffuse tumours, in whom ultrasound was inferior.

The presence of a macroscopic lesion, determined either clinically or by imaging techniques, does not necessarily document accurately the extent of residual tumour. Pathological assessment is important because patients with either a complete clinical or pathological response and no residual macroscopic disease, or marked tumour cell degeneration, have improved survivals (Feldman *et al.*, 1986; Luboinski *et al.*, 1991; Heys *et al.*, 1993*b*). The complete pathological response rate of 18% in our study occurred following chemotherapy and radiotherapy, but no comparable published data are available. However, a complete pathological response rate of 17% has been obtained in operable tumours following similar chemotherapy (Anderson *et al.*, 1991). In their study, primary systemic therapy was used on a selective basis for patients with operable breast cancer of 4 cm or greater in diameter, i.e. chemotherapy was reserved for patients with ER-negative tumours or those in whom endocrine therapy had failed.

The length of follow-up of patients in the current study is insufficient to determine if L-arginine is increasing the disease-free interval or overall survival. Nevertheless, it has been stated that 'pilot studies of potentially more effective approaches to primary chemotherapy need to be pursued' (Rubens, 1992). Our investigation has demonstrated the feasibility of using selective nutrients, albeit in large amounts, in conjunction with chemotherapy. L-arginine is a normal nutrient, and it is cheap, readily available and well tolerated by patients. The high response rates obtained in this pilot study justify further evaluation of L-arginine in a randomised double-blind study.

### References

ANDERSON, E.D., FORREST, A.P., HAWKINS, R.A., ANDERSON, T.J., LEONARD, R.C. & CHETTY, U. (1991). Primary systemic therapy for operable breast cancer. *Br. J. Cancer*, **63**, 561–566.

BONNADONA, G., VERONESI, U., BRAMBILLA, C., FERRARI, L., LUINI, A., GRECO, M., BARTOLI, C., COOPMANS, DE YOLDI, G., ZUCALI, R., RILKE, F., ANDREOLA, S., SILVESTRINI, R., DI FROMZO, G. & VALAGUSSA, P. (1990). Primary chemotherapy to avoid mastectomy in tumours with diameters of three centimeters or more. *J. Natl Cancer Inst.*, **82**, 1539–1545.

CHEUNG, C.W.D. & JOHNSTON, A.E. (1991). Carcinoma of the breast: measurement and the management of treatment. *Br. J. Radiol.*, **64**, 29–36.

DELENA, M., ZUCALI, R., VIGANITTO, G., VALAGUSSA, P. & BONADONNA, G. (1978). Combined chemotherapy–radiotherapy approach in locally advanced ($T_{3b}-T_4$) breast cancer. *Cancer Chemother. Pharamcol.*, **1**, 53–59.

FELDMAN, L.D., HORTOBAGYI, G.N., BUZDAR, A.U., AMES, F.C. & BLUMENSCHEIN, G.R. (1986). Pathological assessment of response to induction chemotherapy in breast cancer. *Cancer Res.*, **46**, 2578–2581.

FORNAGE, B.D., TOUBAS, O. & MOREL, M. (1987). Clinical, mammographic, and sonographic determination of preoperative breast cancer size. *Cancer*, **60**, 761–771.

FOROUHI, P., WALSH, J.S. & CHETTY, U. (1992). Ultrasonography in monitoring breast cancer response to primary adjuvant systemic treatment. *Br. J. Surg.*, **79**, 455.

GROHN, P., HEINONEN, E., KLEFSTROM, P. & TARKKANEN, J. (1984). Adjuvant postoperative radiotherapy, chemotherapy, and immunotherapy in stage III breast cancer. *Cancer*, **54**, 670–674.

HEYS, S.D., SARKAR, T.K., AH-SEE, A.K., HUTCHEON, A.W., EREMIN, J.M. & EREMIN, O. (1993*a*). The management of locally advanced breast cancer: the role of multi-modality therapy. *Surg. Gynecol. Obstet.* (in press).

HEYS, S.D., SARKAR, T.K., AH-SEE, A.K., EREMIN, J.M., BRITTENDEN, J. & EREMIN, O. (1993*b*). Multimodality therapy in the management of locally advanced breast cancer. *J. R. Coll. Surg. Edin.*, **38**, 9–15.

LUBOINSKI, G., NAGADOWSKA, M. & PIENKOWSKI, T. (1991). Preoperative chemotherapy in primarily inoperable cancer of the breast. *Eur. J. Surg. Oncol.*, **17**, 603–607.

MILLER, A.B., HOOGSTRATEN, B., STAQUET, M.D. & WINKLER, A. (1981). Reporting results of cancer treatment. *Cancer*, **47**, 207–214.

PAIN, J.A., EBBS, S.R., HERN, P.A., LOWE, S. & BRADBEER, J.W. (1992). Assessment of breast cancer size: a comparison of methods. *Eur. J. Surg. Oncol.*, **18**, 44–48.

PARK, K.G.M., HEYS, S.D., BLESSING, K., EREMIN, O. & GARLICK, P.J. (1992). The stimulation of human cancers by dietary L-arginine. *Clin. Sci.*, **82**, 413–417.

REMVIKOS, Y., BEUZEBOC, A., ZAJEDAL, A., VOILLMENT, N., MAGDELENAT, H. & POUILLANT, P. (1989). Correlation of pretreatment proliferative activity of breast cancer with the response to cytotoxic chemotherapy. *J. Natl Cancer Inst.*, **81**, 1383–1387.

RODGER, A., JACK, W.J.L., HARDMAN, P.D.J., KERR, G.R., CHETTY, U. & LEONARD, R.C.F. (1992). Locally advanced breast cancer: report of phase II study and subsequent phase III trial. *Br. J. Cancer*, **65**, 761–765.

RUBENS, R.D. (1992). The management of locally advanced breast cancer. *Br. J. Cancer*, **65**, 145–147.

SHIMOSATA, Y., OBOSHI, S. & BABA, K. (1971). Histological evaluation of effects of radiotherapy and chemotherapy for carcinomas. *Jpn. J. Clin. Oncol.*, **1**, 19–35.

SMITH, I.E., JONES, A.L., O'BRIEN, M.E.R., MCKINNA, J.A., SACKS, N. & BAUM, M. (1993). Primary medical (neo-adjuvant) chemotherapy for operable breast cancer. *Eur. J. Cancer*, **29A**, 592–595.

SORACE, R.A., BAGLEY, C.S., LICHTER, A.S., DANFORTH, D.N., WESLEY, M.W., YOUNG, R.C. & LIPPMAN, M.E. (1985). The management of nonmetastatic locally advanced breast cancer using primary induction chemotherapy with hormonal synchronisation followed by radiation therapy with or without debulking surgery. *World J. Surg.*, **9**, 775–785.

SWAIN, S.M., SORACE, R.A., BAGLEY, C.S., DANFORTH, D.N., BADER, J., WESLEY, M.W., STEINBERG, S.M., LIPPMAN, M.E., SORACE, R.A. & BAGLEY, C.S. (1987). Neoadjuvant chemotherapy in the combined modality approach of locally advanced nonmetastatic breast cancer. *Cancer Res.*, **47**, 3889–3894.

TOROSIAN, M.H., MULLEN, J.L., MILLER, E.E., WAGNER, K.M., STEIN, T.P. & BUZPY, G.P. (1983). Adjuvant, pulse total parental nutrition and tumour response to cycle-specific and cycle non-specific chemotherapy. *Surgery*, **94**, 291–299.

TOROSIAN, M.H., TSOU, K.C., DALY, J.M., MULLEN, J.L., STEIN, T.P., MILLER, E.E. & BUZBY, G.P. (1984). Alteration of tumour cell kinetics by pulse total parental nutrition. Potential therapeutic interventions. *Cancer*, **53**, 1409–1415.